# Influence of Wettability and Geometry on Contact Electrification between Nonionic Insulators

**DOI:** 10.1021/acsami.3c05729

**Published:** 2023-06-30

**Authors:** Ignaas S. M. Jimidar, Wojciech Kwiecinski, Gijs Roozendaal, E. Stefan Kooij, Han J. G. E. Gardeniers, Gert Desmet, Kai Sotthewes

**Affiliations:** † Department of Chemical Engineering, Vrije Universiteit Brussel, Pleinlaan 2, 1050 Brussels, Belgium; ‡ Mesoscale Chemical Systems, MESA+ Institute for Nanotechnology and Faculty of Science and Technology, University of Twente, P.O. Box 217, 7500 AE Enschede, The Netherlands; § Physics of Interfaces and Nanomaterials, MESA+ Institute for Nanotechnology, University of Twente, P.O. Box 217, 7500 AE Enschede, The Netherlands

**Keywords:** triboelectric charging, electrostatic interaction, AFM, colloidal probe, contact, electrification, TENGs

## Abstract

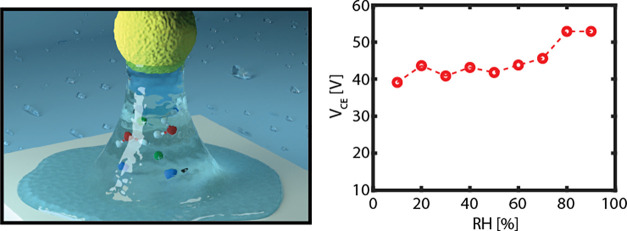

Contact electrification is an interfacial process in which two surfaces exchange electrical charges when they are in contact with one another. Consequently, the surfaces may gain opposite polarity, inducing an electrostatic attraction. Therefore, this principle can be exploited to generate electricity, which has been precisely done in triboelectric nanogenerators (TENGs) over the last decades. The details of the underlying mechanisms are still ill-understood, especially the influence of relative humidity (RH). Using the colloidal probe technique, we convincingly show that water plays an important role in the charge exchange process when two distinct insulators with different wettability are contacted and separated in <1 s at ambient conditions. The charging process is faster, and more charge is acquired with increasing relative humidity, also beyond RH = 40% (at which TENGs have their maximum power generation), due to the geometrical asymmetry (curved colloid surface vs planar substrate) introduced in the system. In addition, the charging time constant is determined, which is found to decrease with increasing relative humidity. Altogether, the current study adds to our understanding of how humidity levels affect the charging process between two solid surfaces, which is even enhanced up to RH = 90% as long as the curved surface is hydrophilic, paving the way for designing novel and more efficient TENGs, eco-energy harvesting devices which utilize water and solid charge interaction mechanism, self-powered sensors, and tribotronics.

## 1 Introduction

Contact electrification (CE) or triboelectric charging is the process of exchanging electrostatic charges when two surfaces are in contact. However, the exact mechanism at the heart of this phenomenon is still under debate. For insulators, even the nature of the charge carrier associated with contact charging has not been settled.^4,48^ Basically, three kinds of charge transfer mechanisms are proposed: (i) electron transfer, (ii) ion transfer,^1^ and (iii) transfer of material.^2^ The reason that a unifying mechanism explaining the tribocharging is missing can be ascribed to the fact that the electrostatic interactions between surfaces are highly complex as they hinge on material,^3,4^ size,^5^ electrical properties, surface properties,^6^ and relative humidity (RH) as well.^7,8^

Understanding the contact electrification mechanism at the micro- and nanoscale is pivotal, as it is currently leveraged in many energy applications, e.g., in triboelectric nanogenerators (TENGs), introduced by the Wang group in 2012,^9,10^ which received great attention as a new energy harvesting application, such as mechanical energy harvesting, self-powered sensing, and tribotronics. Since TENG-based portable and wearable electronic devices will usually operate in varying environmental conditions across the globe, relative humidity is one of the most studied factors that affect tribocharging.^11−14^ The power output is enhanced with increasing RH until reaching a material-dependent optimum that typically lies around 40% RH.^8,13,15^ Above this optimum, the electric output decreases with increasing RH, which can be ascribed to water present at the interface. As the RH changes, water molecules adsorb on the surface, transforming the contact configuration from a single solid–solid interface to a double solid–liquid interface.^1,13,16−22^ Consequently, the charge transfer mechanism also changes; from electron transfer (at solid–solid interactions) to a combination of both electron and ion transfer (at solid–liquid interfaces).^10,23^ Given that water has the ability to charge solid surfaces upon contact, it is thriving as a promising strategy for the massive development of solid–liquid TENGs, droplet-based TENGs, moisture-enabled electric nanogenerators,^24^ and generation of hydrogen peroxide,^25^ to harvest green and renewable electricity from the abundantly present water on Earth.

A key obstacle when studying solid–liquid–solid interacting configurations is that surfaces tend to stick to one another when only hydrophilic surfaces are involved at high humidity levels. Consequently, surfaces can not be released, and the electrostatic charging process at high humidity levels is challenging, if not impossible. To overcome this limitation, we utilize the colloidal probe technique for the first time to investigate the electrostatic interaction induced by CE by immediately measuring (<1 s) the contact electrification voltage between a hydrophilic silica or hydrophobic polystyrene colloidal probe and various hydrophilic uncoated or hydrophobic fluorocarbon-coated substrates as a function of the relative humidity up to 90%. In most material combinations, a clear increasing dependence is observed between the contact electrification voltage and increasing RH. In contrast to previous studies^7,8,13,16,26^ also above the typical optimum of 40–50% RH, an increase in contact charge is observed in this study when the spherical probe is hydrophilic as opposed to when a flat surface in the form of a plateau tip is used. From a fundamental perspective, this is a valuable result as it is indicative of a mechanism in which patches on surfaces contribute to contact electrification,^4,27,28^ as plausibly wet and dry patches are present on the curved colloidal probe leading to an enhancement of the electrostatic interaction between hydrophilic colloidal probes and flat substrates. On the application side, our results are obtained in a similar fashion as contact-separation (CS) operating TENG devices, CS-TENGs, are in agreement with other studies that show that the performance of TENG devices can be enhanced in high humid conditions (RH = 90%) when hydrophobic sliding friction layers in DC TENGs are used,^29^ or cellulose-based surfaces.^30^ In addition, the influence of the contact time on the charging behavior between the two materials is investigated, showing that not only the charge relaxation time constant is dropping with RH but also the time constant of charging. In contrast to room-temperature experiments, measurements performed at elevated temperatures close to the water’s boiling point showed that the charging is constant and lower than that at room temperature. The work presented here explores the contact electrification of a dynamically changing solid–liquid–solid interface and addresses the pressing matter of the influence of surface water on the charging process of DC TENGs, very recently posed by Lyu and Ciampi,^31^ and other energy harvesting applications exploiting the water–solid electrification mechanism.

## 2 Experimental Section

**Figure 1:**
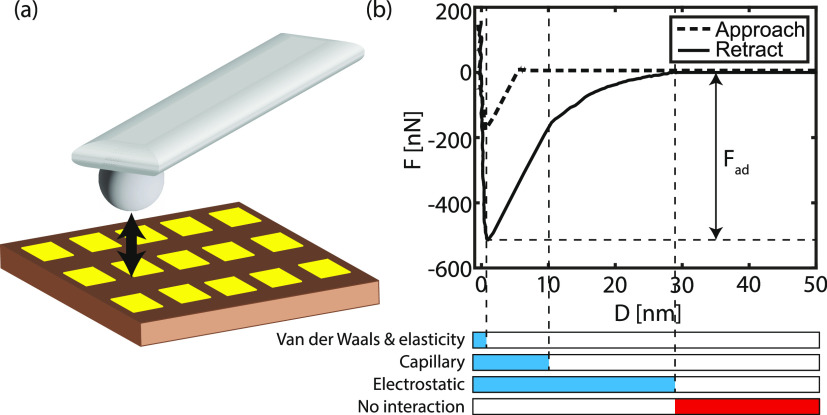
(a) Schematic representation of the colloidal probe method. An AFM cantilever with a colloidal probe is approached and retracted from the substrate resulting in a force–distance (*F*(*D*)) curve. (b) Typical force–distance (*F*(*D*)) curve between a silica colloidal probe and a glass substrate. The blue bars represent the relevant type of interaction force when the colloidal probe has been released at a distance *D* from the flat substrate, while the red bar signifies the range where all interactions vanish.

Force spectroscopy (FS) was performed with a dimension icon atomic force microscopy (AFM, Bruker) to obtain force–distance curves (*F*(*D*)). In this mode, the colloidal probe performs an approach and a retraction cycle at each point on a user-defined grid (see Figure 1a, in most cases 12 × 12). It enables precise control over the applied loading force (*F*_L_), the approach velocity (*v*_a_, which is equal to retraction velocity and cannot be varied independently), and the dwell time (*t*_d_), where the latter two determine the total contact time (*t*_c_) between the colloidal probe and the surface. The measurements were performed with silica and polystyrene colloids with a diameter of 10 μm (NCH-silicon-SPM-sensor with colloidal particle, type: CP-NCH-SiO-D, NanoAndMore). For the parallel plate geometry, a plateau tip with a diameter of 1.8 μm is used (PL2-NCLR, Nanosensors). The substrates used in this study are hydrophilic SiO_2_ and glass (borosilicate glass or Mempax) and hydrophobic CF_*x*_ (2 ≤ *x* ≤ 3, fluorocarbon) coating of 50–75 nm.^3^ The relative humidity was tuned by an in-house-built control setup (Figure S2). The temperature and humidity were measured using a humidity sensor (SENSIRION EK-H4 SHTXX, Humidity Sensors, Eval Kit, SENSIRION, Switzerland), with an accuracy of 1.8% between 10 and 90% RH. Prior to every measurement cycle, the sample was heated at 100 °C in a N_2_ environment to ensure that all water is removed from the surface. Subsequently, the RH was adjusted in the chamber, and we waited for 2–3 h before the experiments were performed. If not indicated within the text, the measurements were performed at room temperature (20 ± 1 °C). The acquired data was processed using a Matlab script, with extracted values for the adhesion force (*F*_a_), snap-out distance (*D*_so_), distance to zero-force (*D*_zf_), indentation (δ), and the contact electrification voltage (*V*_CE_). Two modes were used to measure the tribocharging process with the colloidal probe: the aforementioned force spectroscopy and single-contact charging. In the latter mode, the tip is repeatedly brought into contact at the same location.

More information on materials used in this study and experimental and data analysis routines are included in the Supporting Information.

## 3 Relevant Interaction Forces

Figure 1b depicts a representative force–distance (*F(D)*) curve recorded during the approach and retraction phase of an experiment performed with a silica colloidal probe and a glass substrate. When approaching, the probe jumps into contact with the substrate because the force gradient (mostly the van der Waals force) is larger than the effective elastic constant of the cantilever.^32,33^ During the retraction phase, when the colloidal probe is released from the substrate, the colloid particle experiences different adhesion-type forces, as indicated by the blue bars in Figure 1b. The total adhesion force *F*_ad_ consists mainly of (i) the van der Waals force (*F*_vdW_), (ii) the contact mechanics force (*F*_contact_), (iii) the capillary force (*F*_cap_), and (iv) the electrostatic force (*F*_e_). An animated video is available for a more elaborate explanation of the Supporting Information.

The Hamaker model is used to calculate the van der Waals force through^34,35^
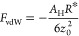
1where *A*_H_ is the Hamaker constant, *z*_0_ ≈ 0.3 nm is the equilibrium separation distance between two smooth bodies, and R* is the reduced radius expressed as (1/R* = 1/*R*_1_ + 1/*R*_2_), with *R*_1,2_ the undeformed radius of bodies 1 and 2, respectively. For the system consisting of a colloidal probe and a flat substrate studied here, R* = *R*.^36^

The contact mechanics force finds its origin in the deformation of two solid bodies at their contact area when brought in contact. The adhesion force (*F*_contact_) of a smooth particle with radius *R* in contact with another surface is expressed as
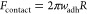
2where *w*_adh_ is the energy change when separating two bodies in contact.

When two hydrophilic surfaces are in close contact, the inevitable water layer at the interface forms a meniscus between the two bodies, even at a low relative humidity.^37^ Consequently, the adhesion force is enhanced and can be described as a capillary force (*F*_cap_),
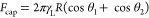
3where γ_L_ is the surface tension of water, and θ_1_ and θ_2_ are the contact angles of the liquid bridge on the two bodies.

All of the distinct forces between two bodies in air are attractive in nature, except the Coulomb force (*F*_e_ or electrostatic force), which can lead to either attractive or repulsive interactions. For the specific contact geometry used in this experiment (cantilever and colloid on a flat surface), the electrostatic force is given by^38^
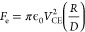
4with *R* the radius of the colloidal particle, ϵ_0_ the vacuum permittivity, and *V*_CE_ the contact electrification voltage, which is the voltage present between the colloid and the surface as a result of the charge accumulation at both surfaces. The configuration geometry can be approximated by the flat plate capacitor model (more information can be found in Section S5 in the Supporting Information). For a more elaborate discussion of these forces, the reader is kindly referred to refs (34−36).

As shown in Figure 1b, *F*_vdW_ and *F*_contact_ are forces that play an important role in force spectroscopy at small distances < 2 nm. Together with the capillary force, these forces mainly determine the maximum adhesion force. For larger separation distances, the capillary and electrostatic forces are important. The moment the capillary bridge snaps *D* ≈ 10 nm in Figure 1b, *F*_cap_ vanishes, and the total force acting on the AFM cantilever only consists of the electrostatic force. Note here that the van der Waals force is also a long-range force still acting on the cantilever, but is of a much smaller magnitude compared to the electrostatic force and can therefore be neglected.^33^

## 4 RH Dependence on the Adhesion and Electrostatic Interaction

**Figure 2:**
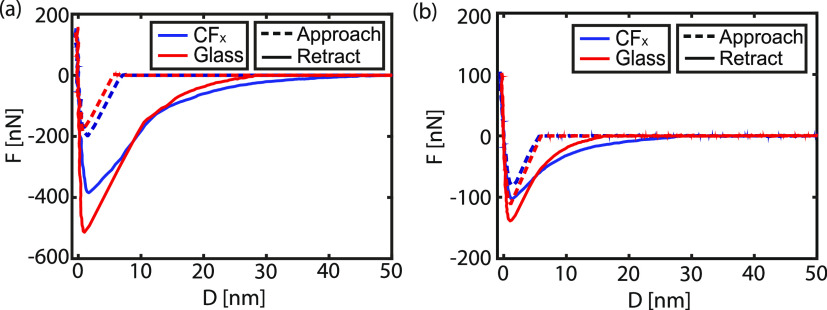
(a) Force–distance (*F*(*D*)) spectroscopy curves of a silica (SiO_2_) colloidal probe on the CF_*x*_-coated (blue) and uncoated glass (red) substrate, respectively. The spectra are acquired at room temperature and 30 % RH. (b) Similar to (a) but here performed with a polystyrene colloidal probe. The dashed (solid) line is the approach (retraction) curve.

Figure 2a shows two typical *F*(*D*) curves for a hydrophilic silica colloid on a hydrophilic glass and hydrophobic CF_*x*_ surface. It can be noticed that the two approach curves obtained on the two different substrates are similar. In both cases, the probe jumps onto the surface. *F*_vdW_ is approximately equal for both surfaces, and therefore the approach curves are almost identical (see Table S2).^34^ Clear differences are observed between the retraction curves obtained on the two distinct surfaces. First of all, the capillary bridge formed between the hydrophilic silica colloid and the glass surface accounts for the higher adhesion measured on the hydrophilic surface. The thin layer of water vapor adsorbed on both surfaces induces the formation of a liquid meniscus that hinders tip detachment from the surface due to the high surface energy. The linear regime (*D* < 10 nm) marks the rupture of the capillary bridge in the retraction curve. This regime is missing in the measurement on the hydrophobic CF_*x*_ surface as a capillary bridge is absent between the hydrophobic surface and the hydrophilic colloidal probe, reducing the adhesion force. Second, a long-range, noncontact interaction is present, which decreases for larger *D*, independent of the surface property. This behavior signifies the presence of a Coulomb force acting between the two surfaces that is induced by the contact electrification mechanism (eq 4). However, as discussed later, the magnitude of the electrostatic interaction is different for glass and CF_*x*_.

From the measurements executed with a hydrophobic polystyrene colloidal probe shown in Figure 2b, it can be inferred that a similar behavior is observed as for the hydrophilic silica colloid on a CF_*x*_ surface. Again the long-range Coulomb force is present after the surfaces make contact, whereas the linear regime is absent from the retraction curves, signifying that no capillary force is present on any substrate due to the hydrophobic nature of at least the polystyrene colloidal probe. The lower adhesion values are explained by the lower Hamaker constant of polystyrene compared to silica (2 × 10^–21^ J and 65 × 10^–21^ J, respectively^35,39^), leading to a lower *F*_vdW_.

**Figure 3:**
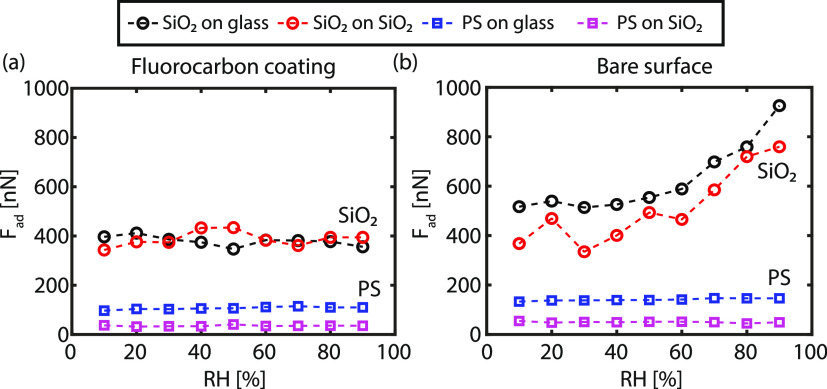
Dependence of the adhesion force (*F*_ad_) on the relative humidity (RH) for a silica (SiO_2_) and polystyrene (PS) colloidal probe on (a) a CF_*x*_-coated SiO_2_ or glass substrate and on (b) a pristine SiO_2_ or glass substrate. The dashed lines connect the data points for clarity. The data including error bars is represented in Figure S9.

As reported in earlier studies,^33,40−43^ the adhesion force increased under more humid conditions for the combination of a hydrophilic surface in contact with a hydrophilic probe. However, *F*_ad_ is independent of the RH for hydrophobic surfaces and hydrophobic probes, or any other combination between a hydrophobic and hydrophilic material. From Figure 3, it is inferred that similar trends can be observed in this study. Only for the combination of a hydrophilic silica colloidal probe on a hydrophilic substrate (SiO_2_ or glass) an increase in *F*_ad_ with RH is found, which can be ascribed to the formation of capillary bridges. The curvature of the meniscus is related to the relative humidity (ρ/ρ_sat_),^44^ therefore both the capillary force and the snap-out distance of the probe from the substrate (*D*_so_ increase, see Figure S6). As soon as one of the materials at the interface is hydrophobic, the bridge formation is suppressed, and the dependence is thus no longer observed.

**Figure 4:**
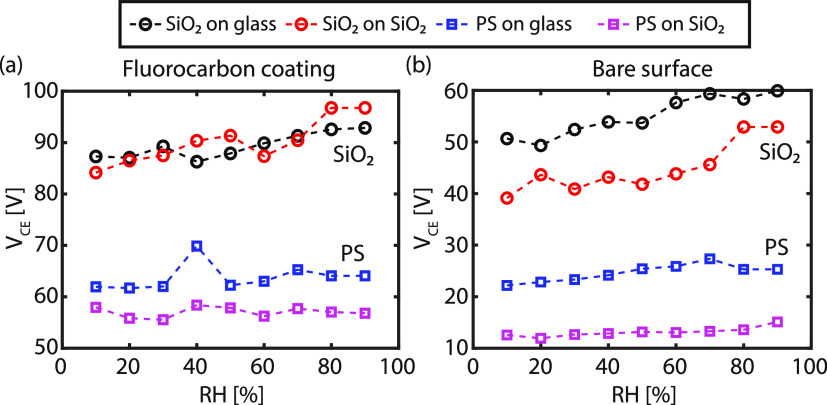
(a) Contact electrification voltage (*V*_CE_) vs the RH between the CF_*x*_-coated SiO_2_ and glass surface and a silica and polystyrene probe, respectively. (b) *V*_CE_ vs the RH between the pristine surface (SiO_2_ or glass) and a silica and polystyrene probe, respectively. The data including error bars are presented in Figure S10.

As already inferred from Figure 2, a long-range electrostatic component induced by tribocharging is present regardless of the wetting properties of the probe or substrate. To quantify the electrostatic interaction between the colloidal probe and the flat substrate, the contact electrification voltage (*V*_CE_) is extracted from the respective *F*(*D*) curves as elaborately described in Section S5 in the Supporting Information. In Figure 4, the contact electrification voltage (*V*_CE_) between the colloidal probe and the respective substrate measured within a time frame of <1 s is plotted as a function of the RH. The largest contact electrification voltage is observed on substrates carrying the CF_*x*_ layer (note the different *V*_CE_ scale in Figure 4), which is in agreement with previous observations where a strong charge accumulation was observed due to triboelectric charging.^3,36^ The CF_*x*_ layer is characterized in the literature as the most negatively charged polymer in the triboseries, thus enhancing the electrification process.^45^ As a result, the CF_*x*_ layer acquires a large negative charge while the silica colloid acquires a large positive charge. A similar mechanism applies to the hydrophobic polystyrene colloid, but the interaction’s magnitude is smaller than the hydrophilic silica colloid, signifying that water possibly has an effect on the electrostatic charging in these two distinct configurations.

The observation that electrostatic charge is exchanged between either type of insulator colloidal probe and the fluorocarbon layer is supported by our previous studies in which Kelvin probe force microscopy (KPFM) measurements performed with a conductive tip show that after rubbing,^3^ or agitation,^36^ the silica or polystyrene microspheres gained a positive charge, while the CF_*x*_ layer charged more negatively. However, in contrast to the colloidal probe measurements performed here, no charge exchange, i.e., electrostatic interaction, between the microspheres and the hydrophilic silicon and glass substrate was previously measured using the KPFM technique. This can be explained by the fact that the colloidal probe technique allows for immediate detection of the electrostatic interaction, whereas the KPFM measurements were performed 10 min after the experiment. Thus, it is implied that the charge dissipates faster on the bare hydrophilic substrates compared to the fluorocarbon-coated substrates. The interested reader is kindly referred to our previous studies where the surface potential maps obtained of the microspheres and flat substrates using KPFM measurements are presented.^3,36,46,47^

Although the empirically established triboelectric series can potentially explain the direction of charge transfer, often deviations are observed,^4,48^ the series fails to explain the dependence on the humidity observed in Figure 4. Note that the relative positions of materials in the triboelectric ladder do not reflect the total amount of charge that can be exchanged at any humidity level.^4^ The electrostatic interaction is enhanced with increasing RH for a hydrophilic silica colloid interacting with a hydrophobic CF_*x*_ layer. A similar trend, albeit weaker charging, is observed if the conditions are inverted (hydrophobic PS colloid interacting with a hydrophilic substrate). However, when contacting two hydrophobic surfaces, no significant change in charging is observed with increasing RH. For two hydrophilic surfaces, on the other hand, an increasing trend in *V*_CE_ is also observed with RH, similar to, but weaker than the hydrophilic–hydrophobic material combinations. The dependence of *V*_CE_ on RH evidently implies that water plays a significant role in the electrostatic interaction between two materials, confirmed in other studies.^7,8,13,18,21,49−51^ Furthermore, the data presented in Figure 4 elucidate that the triboseries should be merely treated as a guideline as the lowest contact electrification voltage is measured between the polystyrene probe and hydrophilic substrates, despite their relative position on the ladder compared to the silica probe and the hydrophilic substrates. Thus, water layers seemingly contain charge carriers that drive the onset of electrostatic attractions, which is plausibly affected by the wettability and curvature of the substrate, i.e., the water layers’ structure on the surface.

## 5 Charging Mechanism of Hydrophilic and Hydrophobic Surfaces When Water Is Present

During the contact-separation event of the colloidal probe on the substrate, the interface between them dynamically transitions from solid–liquid–solid (approach phase) to solid–solid (in contact) and again to a double solid–liquid interface (retract phase), signifying the complexity of the contact electrification mechanisms at work in our experiments. When a water layer is in contact with another surface, it has been proposed that, in general, two transfer mechanisms play an important role at the solid–liquid interface: (i) ion adsorption^1,7^ and (ii) electron transfer.^22^ The electron transfer process is explained by a so-called “two-step process”.^52^ In the first step, molecules and ions present in the liquid impact the solid such that electrons from the water molecules are transferred due to the overlap of the electron clouds of the solid atoms and the molecules.^53^ This ionization reaction at the solid–liquid interface generates both electrons and ions on the solid surface, while ions from the liquid can also be adsorbed on the surface, leading to a charged solid surface.^52^ In the second step, the mobile cations in the liquid and the freely migrating ions pushed from the solid surface will be attracted to migrate toward the charged surface by the electrostatic interactions, forming an electric double layer.

Note that the electron transfer and ion adsorption processes simultaneously occur at the solid surface and depend on the amount of water present on it. Which of these processes will dominate depends on the electron-capture and adsorption capabilities of the respective surfaces. Typically, polymers with fluorine groups (in this case CF_*x*_) can directly receive electrons from the impacting water molecules and adsorb anions from the liquid. Consequently, when separating the probe from the surface, the most hydrophobic surface remains negatively charged, which is in line with our previous KPFM studies,^3,36,46^ while the freely migrating cations remain in the water layer due to the higher mobility. Thus, given that we measure an electrostatic attraction between the colloidal probe and the solid surfaces, it can be safely concluded that due to asymmetric surface properties, one surface with the most affinity for electron-capturing charges negatively, while the other surface remains with water layers charges positively.

When two hydrophobic surfaces are brought into contact, the net charge transfer is determined by the asymmetric coverage of water islands between the two contacting surfaces, the likelihood for an ionization reaction and the affinities for adsorbing anions for different materials.^16,20,22^ As the water layer thickness on the hydrophobic materials is independent of the RH (also supported by the constant adhesion vs RH in Figure 3a), and both transfer processes rely on the presence of water, no change is expected in the charge distribution on both surfaces. Therefore, no charging dependence is expected and observed with RH between two hydrophobic materials, the PS probe and fluorocarbon layer, studied here (cf. Figure 4a). In a recent study by Lin et al., it was concluded that electron transfer is the more dominant charge transfer method between two hydrophobic surfaces, i.e., on a solid–solid interface.^22^ However, because the charge is measured just after the contact electrification process, no distinction can be made between the two processes.

The situation immediately alters when the hydrophilicity of one of the surfaces is changed. The amount of water on the hydrophilic surface is much higher compared to the hydrophobic ones. When contacted, a liquid bridge is formed between the two surfaces, and the interfaces of both surfaces are fully wetted.^54^ When the two surfaces are again separated, the hydrophobic surface has a higher affinity for negatively charged carriers, while the cations remain in the water layer present on the hydrophilic surface. As the water layer thickness on a hydrophilic surface heavily depends on the RH, the number of mobile charges within the system varies. Therefore, changing the relative humidity significantly affects the contact potential between a hydrophilic and hydrophobic surface. The charging observed for the silica colloid interacting with a CF_*x*_ surface as a function of RH (Figure 4a) is stronger compared to charging between a polystyrene colloidal probe and a SiO_2_ or glass substrate (Figure 4b), although both material combinations are described as a hydrophobic–hydrophilic system. This is explained by the higher hydrophobicity^13^ of the CF_*x*_ layer in comparison with polystyrene. As a consequence, more negatively charged ions are attracted toward the CF_*x*_ layer, increasing the amount of charge and thus a more considerable contact electrification voltage. The difference in electrostatic interaction measured between a polystyrene colloidal probe and the SiO_2_ and glass substrate can be ascribed to the difference between the substrate contact angles in contact with water (36 vs 10°, respectively).

Surprisingly, when two hydrophilic materials are brought into contact, a dependence on the RH is also found (see Figure 4b). Based on the contact angles of water on the materials, a small amount of electrification on the surfaces is expected.^20^ However, similar to the silica colloid and fluorocarbon-coated substrate case, the electrostatic interaction is enhanced by a geometrical asymmetry of the two surfaces in contact: a planar substrate and a spherical colloid. Previous experiments showed that water films on particle surfaces are not continuous even for very hydrophilic curved surfaces.^19,55^ This leads to a similar scenario that describes the interaction between a hydrophobic probe and a hydrophilic surface. Dry patches are present on the colloid, and more ions are available on the planar surface. This result supports the notion among scientists that a surface locally contains acceptor/donor patches that contribute to the contact electrification mechanism.^4^

As there is solid evidence that material transfer can drive contact electrification,^56,57^ we cannot exclude that material transfer contributes to the charge transfer, albeit little, when the probe and substrate are in contact, i.e., during the solid–solid interactions. The latter will particularly apply to the case of the PS probe in contact with fluorocarbon surfaces. Despite this evidence, we want to emphasize that the electrification enhancement observed here with increasing RH can primarily be ascribed to the presence of increasing water content between contacting surfaces.

### 5.1 Influence of Temperature and Geometry

**Figure 5:**
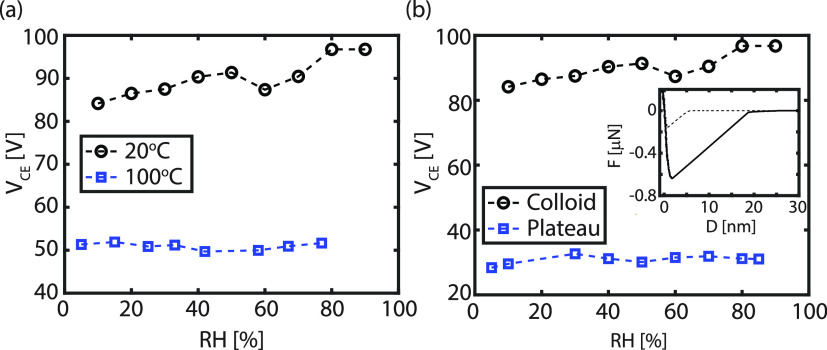
(a) Contact electrification voltage (*V*_CE_) vs the RH between the CF_*x*_-coated SiO_2_ surface and a silica colloidal probe when the surface is kept at a temperature of 20 and 100 °C. The black line is the same data set as depicted in Figure 4. A clear difference is observed when the temperature is raised near the boiling point of water, removing the water component from the system. (b) *V*_CE_ vs RH between the CF_*x*_-coated SiO_2_ surface and a silica probe with a spherical (10 μm colloidal probe) and a flat (1.8 μm plateau tip) geometry. No dependence is observed between the contact electrification voltage and RH when a plateau tip is used. Inset: Characteristic *F*(*D*) curve for a plateau tip on CF_*x*_ surface.

To corroborate that the presence of water indeed influences the triboelectric charging process, the experiment performed using a silica colloid on a CF_*x*_ surface is repeated at elevated temperatures. In Figure 5a, the dependence between the contact electrification voltage as a function of RH is shown for different temperatures. In this particular case, it is previously shown that the *V*_CE_ value increases when the humidity level in the chamber rises (cf. Figure 4a) at room temperature. However, when the temperature of the substrate is raised to 100 °C, *V*_CE_, and thus the electrostatic interaction remain constant while the RH is varied. Thus, this confirms that the presence of a water layer has a strong effect on the triboelectric charging process. At 100 °C, the majority of the absorbed water is removed from the substrate and therefore cannot contribute any longer to the ion exchange process.

Not only the dependence on the RH but also the absolute value of *V*_CE_ is altered with temperature, implying that water contributes to a stronger electrostatic attraction between the probe and substrate. Previous studies have already observed a drop in the charge transfer rate with temperature.^53,58^ This observation is well explained by the electron cloud-potential well model.^53^ Prior to the contact between the probe and the surface, the electron clouds of both surfaces remain separated without overlap. The potential well prevents the electrons from freely escaping the material, which is the case for nonconducting materials. When the two materials are in contact, the two potential wells merge into an asymmetric double-potential well in which electrons can transfer from one material to the other. Note that at these elevated temperatures, the configuration corresponds to a solid–solid interface as the surfaces touch at a dry contact.

It is key that TENG devices can perform superior under the high level of environmental humidity;^14,59^ however, most studies find that charging, and thus their performance drop beyond RH < 40%.^7,8,13,26^ However, a few strategies are currently investigated to keep a normal and steady function of TENG devices for high-RH conditions by preventing water in the device using encapsulating technology^59^ or even more hydrophobic friction layer in a sliding mode operating TENGs.^14,29^ Another strategy that has proven to successfully enhance the charge induced by CE is the use of cellulose or starch films in which water molecules can form hydrogen bonds with hydroxyl-rich biomaterials.^30,59^ Similar to those studies, we found an enhancement of the contact electrification process with increasing relative humidity found in Figure 4 after contacting and separating a colloidal probe from the substrate, even for the hydrophilic–hydrophilic combination (silica–silicon).

The main difference between the current study and standard TENGs is the geometrical asymmetry present comprising a planar substrate and a spherical colloid. To justify this hypothesis, a similar experiment to that in Figure 4 is performed, however, with another probe geometry. Figure 5b shows the result obtained using a 1.8 μm silica plateau tip (cf. Figure S1c,d), which mimics the conventional parallel plate configuration used for TENGs. While an increasing contact electrification voltage with RH is observed for the colloidal probe geometry, *V*_CE_ is independent of RH for a plateau tip. Water films on curved surfaces are never continuous, even for very hydrophilic surfaces, which reduces conductivity and the charge to be transferred to the external environment.^13^ Moreover, as a full monolayer of water is present on the plateau tip’s surface (which is the case for flat surfaces), no wet and dry patches are present anymore, reducing the charge exchange within the system (see Figure S9). Therefore hydrophilic curved surfaces are much more affected by the RH conditions as the number of wet and dry patches increases. Based on this observation, producing TENGs with an asymmetric geometry may be a promising avenue to develop devices that also work in humid environments. Additionally, the enhancement of interface charging up to RH = 90% in a solid–liquid–solid asymmetric system may appeal to those working in the area of liquid–solid and droplet-based TENGs in which energy is harnessed by the propensity of water to charge surfaces.^60−63^

For the flat plate geometry, no RH dependence is observed, although other studies show an enhancement of the power output with a maximum around RH = 40%.^7,8,13,16,26^ This discrepancy is most likely caused by the active contact area of the system and the roughness of both surfaces. Due to the microscopic dimensions of the experiment and the nanoscopic roughness of both the plateau tip (Figure S2c,d) and the surface (Figure S4), the influence of roughness on the experiment is mostly suppressed. In macroscopic designed TENGs, roughness plays a major role in the tribocharging process because more water bridges can form in the gaps which enhances the charge transfer.^13^ Another study found a continuous decrease of the transferred charge with increasing humidity.^11^ In that study, the contact geometry consists of two pyramidal patterned surfaces, again stressing the importance of the contact geometry and roughness of the surfaces on the RH-dependent charging behavior.

### 5.2 Single-Contact Charging

The absence of charge effects in the approach curves indicates that triboelectric charging and discharging occur on a shorter time scale. In order to investigate the time scale with which charging and discharging occurs, additional measurements have been performed with different approach and retraction velocities (*v*_a_) as well as varying dwell times (*t*_d_) (see Section S3 in the Supporting Information for more information). Both parameters influence the contact time (*t*_c_), which is the total time the colloidal probe and the surface can exchange charge across their interface in contact. Therefore, the contact time mainly depends on *t*_d_ and is only influenced by *v*_a_ at low velocities. Note that in the employed experimental strategy, the contact electrification voltage is measured only after a certain separation (*D* ≈ 20 nm) between the silica probe and substrate is reached, implying that the probe has already discharged some of its gained charge to the surroundings, particularly at low retraction velocities.

**Figure 6:**
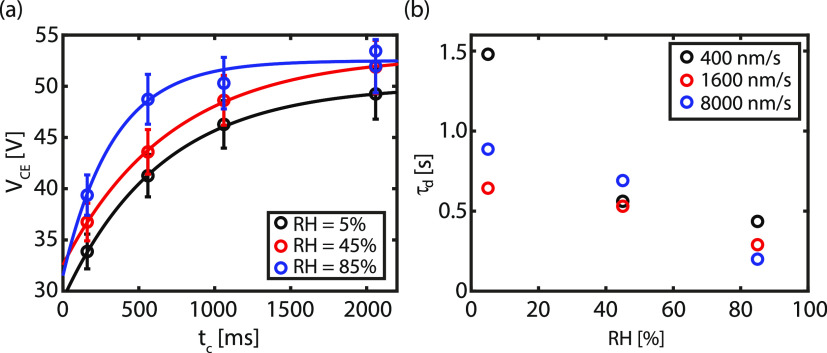
(a) Contact electrification voltage (*V*_CE_) vs contact time (*t*_c_) for different relative humidities at a retraction velocity of *v*_a_ = 1600 nm/s for a silica colloid in contact with the CF_*x*_ layer. The solid line is the model described by eq 5. Standard deviations are based on the averages of at least five independent experiments. (b) Extracted charging time constant (τ_d_) for different retraction velocities as a function of relative humidity.

In Figure 6a, the contact electrification voltage is determined as a function of contact time for different relative humidities between a silica colloidal probe and the CF_*x*_. A clear trend is observed in which *V*_CE_ increases for longer contact times in contrast to the adhesion force.^64^ For the other material combinations, the charging process is too fast (pristine SiO_2_) or weak (polystyrene probe) to observe the same trends. The charging process as a function of time is described by^65^
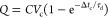
5with *Q* the contact charge, *C* the electrical capacity, *t*_c_ the contact time, τ_d_ the time constant of charging, and *V*_c_ the potential when in contact. As *V*_CE_ ∝ *Q*/*C*, hence, eq 5 can be applied to the experimental data depicted in Figure 6a. An excellent agreement is obtained between the data and the model described in eq 5.

In addition, the charging process depends on the relative humidity, as larger contact electrification voltages are observed for increasing RH. From the fit, the time constant of charging can be extracted. τ_d_ is plotted for different retraction velocities as a function of RH in Figure 6b. Again a declining trend is observed, where the charge time constant decreases with increasing RH, indicating that triboelectric charging is faster in higher humid conditions as a result of the high mobility of the ions present in water. This observation is similar to other studies where the discharging process occurs faster at higher RH due to a higher surface conductivity.^66−68^ At low RH, small patches of water are present on the hydrophilic colloidal probe, while there is barely water on the hydrophobic substrate, leading to minimal charge diffusion. The water patches become larger and more connected with increasing RH, leading to a larger covered area which enhances the surface conductivity. Consequently, a lower charging constant is obtained. A caveat here is that the time constants obtained at the lower retraction velocities should be interpreted with caution, as the time constant entails a discharging part that is dominant when retraction velocities are low (see Section S8 in the Supporting Information). On the other hand, when the retraction velocity is increased, i.e., the measurement is performed faster, a higher contact electrification voltage is measured, as shown in Figure S11 in Section S8 in the Supporting Information. Thus, if the electrostatic interaction was measured at later timescales, no charge transfer would possibly be measured at higher relative humidity levels, as is the case in other reports. This observation underscores the advantage of the colloidal probe technique to measure the charge transfer due to contact electrification rapidly.

The values found for τ_d_ are much higher compared to the charge transfer rate (by means of electrons) occurring during tribocharging between metals (seconds vs microseconds).^69^ This observation can be directly ascribed to the presence of a water layer. When the probe and the substrate are in contact, the anions and cations have to diffuse and migrate through the water bridge in order to rearrange themselves. As already discussed, these ions have a higher mobility than the water molecules.^17,70^ Thus, the mobility of ions across the interface affects the charging rate.

In contrast to other studies,^20^ no additional charging is observed between multiple contacts (see Figure S10). The measured contact electrification voltage remains constant regardless of the number of contact events, similar to the observation that the approach curve shows no sign of charging (see Figure 2). This is explained by the low charging constant. The time between successive measurements is approximately 1 s, providing ample time for the charge to diffuse away. Only when the approach and retraction speed is increased significantly (*v*_a_ > 8000 nm/s), an electrostatic interaction between the probe and the surface is also observed in the approach curve (see Figure S12). However, an additional increase in the contact electrification voltage as a function of the number of touches remains absent.

The colloidal probe configuration exploited here is a straightforward, fast, and direct way to control and monitor humidity effects, allowing for examining the contact electrification at higher humidity. In addition, the colloidal probe technique allows distinguishing between the different forces involved in the CE process; see Figures 3 and 4. When performed in a liquid environment, the capillary force vanishes even further, enhancing the contribution of the electrostatic and van der Waals forces. Also, the charging process itself is accessible (see Figure 6) in contrast to other experimental methods where only the final amount of charge can be detected, such as in a Faraday cup, a parallel plate setup, or Kelvin probe force microscopy.^3,4,20,46,69,71−73^ On the other hand, the transient process until the saturation charge is acquired and its polarity remains unknown or difficult to quantify within the colloidal probe measurements.

Furthermore, it has been recently reported that the green energy source hydrogen^74^ or hydrogen peroxide^25^ can be directly produced from moisture present in air already at low relative humidities. As shown in this work, the colloidal probe method is a suitable platform to study the effects of humidity and may be included in an electrochemical setup to study the effect of mechanical forces on harvesting hydrogen from air. The same holds for moisture-enabled electric nanogenerators, where charge exchange occurs between the humid environment and different surfaces to harvest the generated energy.^24^ From the results obtained in this report, we can infer that the colloidal probe technique can be an excellent candidate for studying the charging process in moisture-enabled electric nanogenerators.

## 6 Conclusions

We have shown that distinct interactions exist between different combinations of colloidal probes and substrates, namely, (i) hydrophilic–hydrophilic, (ii) hydrophobic–hydrophilic, (iii) hydrophilic–hydrophobic, and (iv) hydrophobic–hydrophobic, as a function of relative humidity. As expected, the capillary force dominates the adhesion between a hydrophilic silica probe and a hydrophilic substrate, whereas the adhesion remains approximately constant as a function of the relative humidity as soon as a hydrophobic material is involved. As the RH increases, in three of the four cases (situations i, ii, and iii) a clear increasing dependence is observed in the contact electrification voltage. These findings confirm the influence of humidity, and especially the presence of anions and cations in water, on the charging process between two materials, specifically nonionic insulators. Our results confirm that electron transfer is the primary mechanism for contact electrification for the hydrophobic–hydrophobic combination, as no change in contact electrification voltage is measured with varying humidity levels.

The colloidal probe configuration enables examining the contact electrification in <1 s at a higher relative humidity (RH > 40%) because of the controlled retraction mechanism, which reduces the influence of capillary forces. This allows studying the charging process itself, revealing that the charging time constant is strongly decreasing with increasing relative humidity, similar to the charge relaxation time constant. In addition, we show for the first time that the curved surface (of the colloidal probe) enhances the charging process between the surfaces because wet and dry patches are present on curved surfaces (even at high relative humidity), which drives the ion exchange. We envision that the colloidal probe technique can serve as a promising platform in studying the charging process and the concomitant development of more efficient miniaturized energy harvesters, e.g., various adaptations of TENGs, needed in our collective effort to transition towards a green industry.

## Supporting Information Available

Additional experimental details; materials; method and acquire sequence; equation derivation; error analysis and the influence of multiple touches; and the approach velocity on the contact electrification voltage (PDF)
Colloidal probe_procedure (MP4)
