# Supplementary material for: Influence of Wettability and Geometry on Contact Electrification between Nonionic Insulators
Source: ACS Appl Mater Interfaces. 2023 Jun 30;15(35):42004–14. doi: 10.1021/acsami.3c05729 (PMC10485807; doi:10.1021/acsami.3c05729)
Supplement: Supplementary file 1 — am3c05729_si_001.pdf [file am3c05729_si_001.pdf]

Supporting Information: Influence of Wettability and Geometry on Contact Electrification Between Nonionic Insulators

Ignaas S. M. Jimidar,[*,†,‡] Wojciech Kwiecinski,[¶] Gijs Roozendaal,[¶] E. Stefan Kooij,[¶] Han J.G.E. Gardeniers,[‡] Gert Desmet,[†] and Kai Sotthewes[*,¶]

†*Department of Chemical Engineering, Vrije Universiteit Brussel, Pleinlaan 2, 1050 Brussels, Belgium*

‡*Mesoscale Chemical Systems, MESA+ Institute for Nanotechnology and Faculty of Science and Technology, University of Twente, P. O. Box 217, 7500AE Enschede, The Netherlands*

¶*Physics of Interfaces and Nanomaterials, MESA+ Institute for Nanotechnology, University of Twente, P.O. Box 217, 7500AE Enschede, The Netherlands*

E-mail: i.s.m.jimidar@utwente.nl; k.sotthewes@utwente.nl

Contents

1 Sample preparation **S3**

2 Probe and sample parameters **S3**

3 Relative humidity setup **S4**

4 Acquirement of the $F(D)$ curves **S5**

5 Electrostatic force extraction **S7**

6 Snap-out distance, distance to zero force and indentation **S10**

7 Error Analysis **S12**

8 Influence of multiple touches **S13**

9 Influence of the approach velocity **S15**

References **S19**

1 Sample preparation

Silicon wafers covered with a 2 nm native oxide, and borosilicate glass (Mempax®) wafers were patterned with a hydrophobic fluorocarbon coating using a standard protocol in the MESA+ Institute for Nanotechnology of the University of Twente. Firstly, the wafers were primed by spin coating (4000 rpm, 30 s) HexaMethylDiSilazane (HMDS), followed by spin coating (4000 rpm, 30 s) a positive photoresist (Olin OIR 906-12) on the wafer. Hereafter, using a UV light source ($350-450$ nm), the photoresist on the wafer was illuminated through a mask with the geometrical patterns (EVG® 620 Mask Aligner). After the exposure, the resist was developed by placing the substrate for 60 s inside a beaker with the developer (OPD4262). The substrate was rinsed with DI water until the conductivity of the water reached 10 MΩ to remove all residues of chemical agents. Once the substrates had been dried, the CF_x-layer ($2 \leq x \leq 3$) was deposited on the substrate by plasma polymerization of CHF_3 in a reactive ion etcher (RIE) system (25 sccm CHF_3, 11W, 130 mTorr, 8 min., electrode temp. 20 °C). Finally, using a lift-off process (substrate submerged in acetone sonicated for 15 min, followed by 15 min of sonication in IPA), all residues of the resist were removed from the substrates. Subsequently, the wafers were again rinsed with DI water to remove all chemical agent residues from the substrates, until the conductivity of the water reached 10 MΩ.

2 Probe and sample parameters

In Table S1 and S2 the material properties of the probes and samples are summarized. The radius of the colloidal probe is checked using a SEM (see Fig. S1). The spring constant is determined by using the thermal vibration method.[1] First the deflection sensitivity is determined on a sapphire sample and subsequently the spring constant is measured from the thermal tuning.

Table S1: Various cantilever properties including the Young's modulus (Y), poisson ratio (ν), spring constant (k), resonance frequency (f_r, tip radius (r), relative permittivity (ϵ_r), particle density (ρ_p) and resistivity (ρ_R) . The spring constant is determined using the thermal vibration method.[1]

	Y [GPa]	ν	k [N/m]	f_r [kHz]	r [μm]	ϵ_r	$\mathrm{p_p}$ [kg/m^3]	ρ_R
SiO_2 colloid	169[2]	0.358[2]	20-50	204-497	10.2	11.7[3]	1850	10^{14}
Polystyrene colloid	3.3	0.34	20-50	204-497	10.2	2.5	1050	5×10^{14}
SiO_2 plateau	169[2]	0.358[2]	20-50	164-236	0.9	11.7[3]	1850	10^{14}

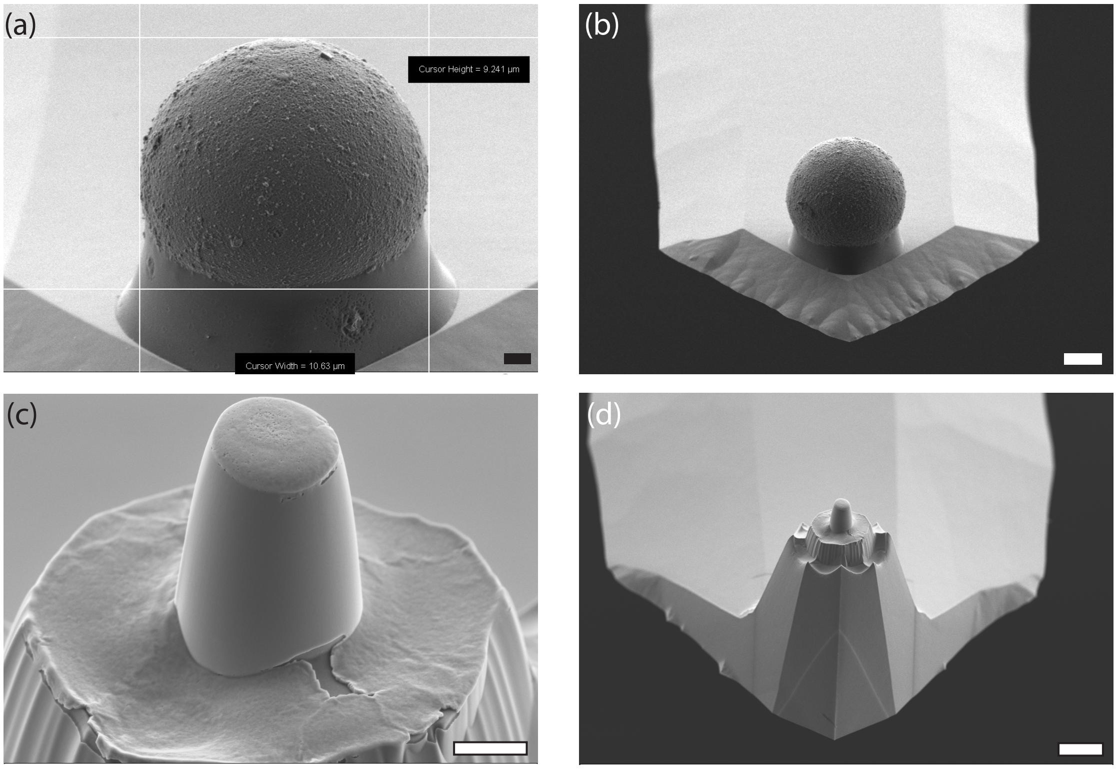

Figure S1: SEM images of the different probes used in this study. (a-b) Two 10 μm colloidal probes. (a) scale bar 1 μm, (b) scale bar 2 μm. Two 1.8 μm diameter plateau tip. (c) scale bar 1 μm, (d) scale bar 5 μm.

3 Relative humidity setup

In Fig. S2 a schematic representation of the relative humidity controller is shown. Dry nitrogen vapor is flowing through two tubes: (i) directly towards the buffer chamber and

Table S2: Various bulk material properties including the Young's modulus (Y), poisson ratio (ν), contact angle (θ), relative permittivity (ϵ_r), density (ρ_m), Hamaker constant (A_H) and surface energy (γ).

	Y [GPa]	ν	θ [°]	ϵ_r	ρ_m [kg/m^3]	A_H [10^{-21}J]	γ [N/m]
Silicon	700[2]	0.358[2]	70[4]	11.7[3]	2650	36[5]	0.05[5]
Mempax®	64	0.2	10[4]	4.5	2200	115[6]	0.28[6]
CF_x	0.44	0.27	110[4]	3.43	1350	62[7]	0.02[7]

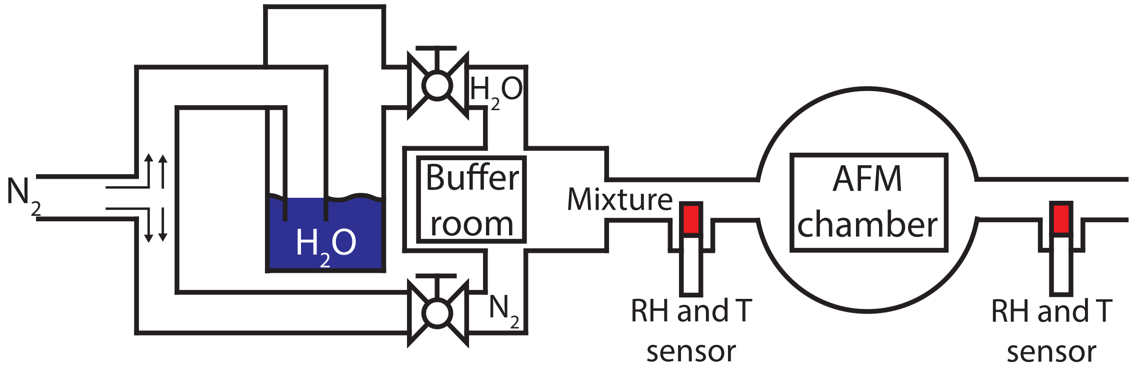

Figure S2: Humidity control setup.

(ii) through water in order to make water vapor. With the two manual valves, the relative humidity can be adjusted. A buffer chamber is inserted to make sure both the H_2O and N_2 mix well. Just behind the buffer chamber, a relative humidity sensor is located. A similar sensor is placed behind the AFM chamber, to verify if the relative humidity remains the same throughout the whole process.

4 Acquirement of the $F(D)$ curves

In Fig. S3 an example is given of the measured force (F) as a function of time (t). The approach (t_a) and retraction (t_r) time are determined by the approach velocity (v_a). First the probe is out of contact and no force is exerted on the probe. At a certain distance from the surface, a total force is acting on the probe which consists of a combination of the van der Waals force (F_{vdW}), the electrostatic force (F_e) and the capillary force (F_c). When water is present on the surface or probe, the snap-in event is more pronounced compared to the other

two forces (and depends also on the stiffness of the cantilever). From the snap-in event, the probe is into contact with the surface. The tip is pushed onto the surface till the load force (F_L) is reached.[8,9] The load force remains constant through all the experiments. From this point the approach procedure is finished and the dwell time starts (t_d). During the dwell time, the force is kept constant. When the dwell time is over, the retraction sequence starts. The probe is lifted till it snap-out of contact. From this moment the probe is no longer in contact with the surface, but long-range forces still act on the probe (such as F_e). When the probe is retracted even further away from the surface, the force acting on the probe is reaching zero and the procedure is over.

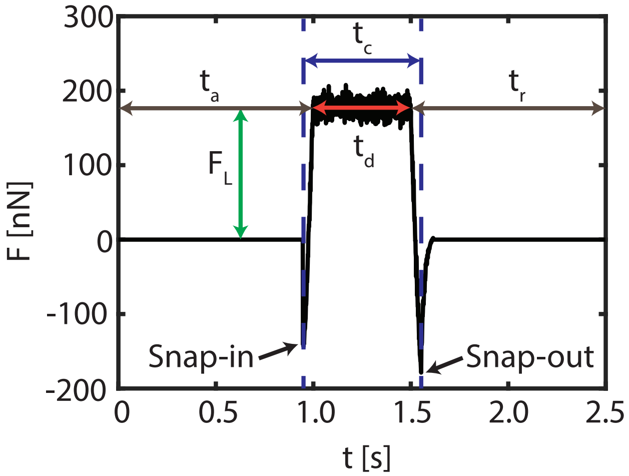

Figure S3: Example of a Force versus time curve. The contact time (t_c) consists of the dwell time (t_d) and the approach velocity ((v_a), which determines approach (t_a) and retraction time (t_r). The force applied to the colloidal probe during contact is given by (F_L)

.

The contact time (t_c) is the time between the snap-in and snap-out event. It is influenced by the dwell time (t_d) and the approach velocity (v_a). The approach velocity determines the time between the snap-in event and the moment the maximum force is acting on the probe, and the time between the end of the dwell time and the snap-out event.

When a matrix measurement is performed, the probe touches the surface in a grid-like fashion. Both the CF_x and the pristine surface are measured simultaneously (see Fig. S4). On the interface between the CF_x layer and the Si wafer, $F(D)$ measurements are performed

in a matrix format. In the inset of Fig. S4(b) an example is depicted. Every square is a measurement and when a force distance curve is performed, the probe moves to the next square till the whole area is covered. When finished, force-distance curves are collected on both the CF_x layer as well as on the pristine Si surface. Note here, that only one measurement is performed per position. The median curve is than extracted from all the curves on the same surface.

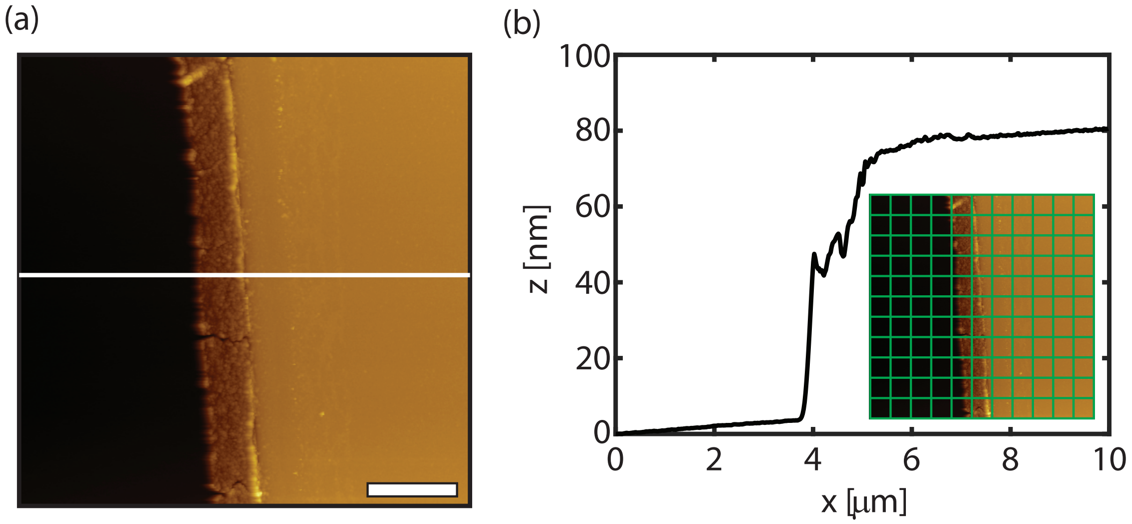

Figure S4: (a) Topography image of the CF_x coated zone on a Si wafer (10 × 10 μm, scale bar 2 μm). (b) Cross-section height profile along the white line in (a). The CF_x layer is approximately 80 nm thick. Inset: A matrix indicating where the probe performed $F(D)$ experiments.

For single point measurements, a force-distance curve is performed multiple times on the same position. In this procedure, the history of measurements affects the result, as charging of the colloidal probe and the substrate changes the outcome of the force-distance curves. This procedure allows for studying the charging process as a function of the number of touches.

5 Electrostatic force extraction

In order to extract the contact electrification voltage (V_{CE}) from the measurements, the electrostatic force component needs to be extracted from the $F(D)$-curves. The electrostatic

force acting on the probe and cantilever is described by Law and Rieutord[10,11] and is given by

$$F_{\mathrm{e}} = \pi\epsilon_0 V_{\mathrm{CE}}^2 g(D) \tag{S1}$$

where ϵ_0 is the vacuum permittivity and g(D) the geometrical factor as a function of distance (D). The geometrical factor consists out of three components, the apex, cone and cantilever (for more information, see ref.[11]). All three components contribute in different ways and have different distance dependencies. Due to the different components, it is regarded as a difficult task to quantify electrostatic forces. Therefore a useful approximation for all distances is

$$F_{\mathrm{e}} = \pi\epsilon_0 \frac{R^2 V_{\mathrm{CE}}^2}{D(D+R)} \tag{S2a}$$

$$F_{\mathrm{e}} = \pi\epsilon_0 V_{\mathrm{CE}}^2 \left(\frac{R}{D}\right) \text{ for } R \gg D \tag{S2b}$$

$$F_{\mathrm{e}} = \pi\epsilon_0 V_{\mathrm{CE}}^2 \left(\frac{R}{D}\right)^2 \text{ for } R \ll D \tag{S2c}$$

with R the radius of the tip or colloidal particle. For probes with a large radius, such as colloidal probes, equation S2 is applicable. On short distances, both the electrostatic as well as the van der Waals force are present, however, typically the electrostatic force is of a much larger magnitude compared to the van der Waals force.[12]

As depicted in Fig. 1 in the main text, multiple forces constitute the colloid probe-surface interaction. In order to extract the V_{CE} value, first the electrostatic component in the $F(D)$ has to be determined. An example is shown in Fig. S5. Especially at high RH, the capillary force significantly affects the shape of the $F(D)$-curve, making it difficult to determine the starting point of the electrostatic force. In order to determine where the electrostatic force is more dominant compared to the capillary force, the derivative and second derivative of

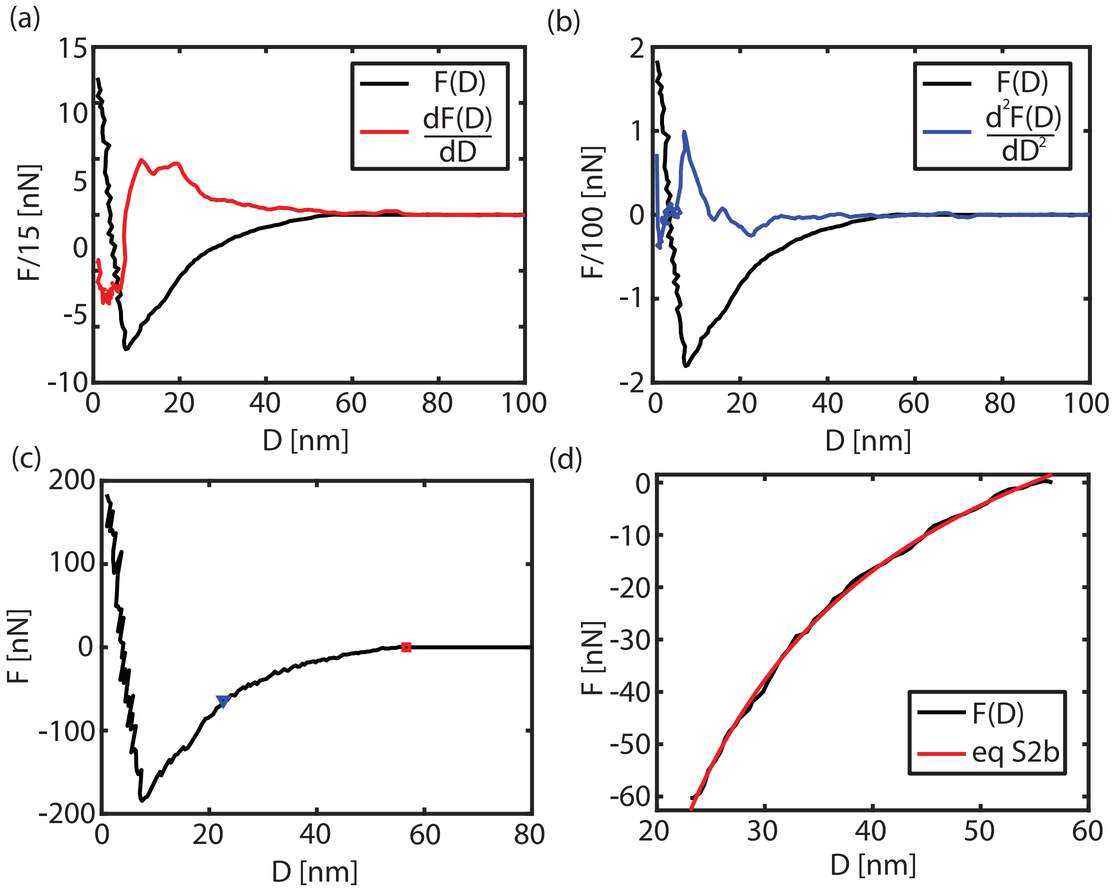

Figure S5: (a) A $F(D)$-curve (black) at RH $= 45\%$, $t_\mathrm{d} = 2000$ ms and $v_\mathrm{a} = 400$ nm/s and the derivative of the $F(D)$-curve (red). The same $F(D)$-curve (black) as in (a) and the second derivative of the $F(D)$-curve (blue). (c) The same $F(D)$-curve as in (a,b) with markers which indicate the begin (blue triangle) and end (red square) of the electrostatic force component. (c) The electrostatic part of the $F(D)$-curve in (b). The red line is a fit based on equation S2.

the $F(D)$ are extracted. For low RH values, the first derivative is sufficient to determine the starting point, as this coincides with the highest value in the derivative. At higher RH values, no peak but a plateau is present in the $dF(D)/dD$-curve (see Fig. S5(a)). Therefore the minimum in the $d^2F(D)/dD^2$-curve is determined. This is the point where the slope changes in the $F(D)$-curve (blue triangle in Fig. S5(c)). The end point is placed at the position where the force reaches zero (red square in Fig. S5(c)). The data in between these points is then used to fit eq.S2, from which the V_{CE} is extracted.

6 Snap-out distance, distance to zero force and indentation

Besides the obtained V_{CE} and F_{ad} in the main text, several other parameters can be extracted from the obtained F(D) curves. The snap-out distance (D_{so}) is the distance at which the water meniscus snaps (i.e. the point where the electrostatic force starts to dominate, or the blue triangle in Fig. S5). In Fig. S6 the snap-out distance is plotted as a function of RH. Only for the hydrophilic-hydrophilic material combination a D_{so} is extracted, as the liquid bridge is not or barely present when a hydrophobic material is involved.

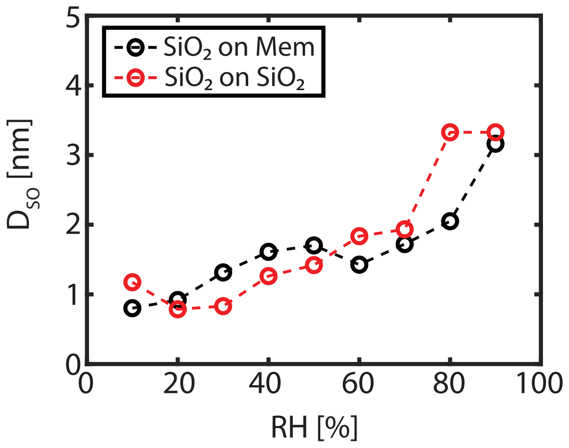

Figure S6: The Snap out distance (D_{so}) as a function of the relative humidity. A similar increasing trend with increasing RH is found as for the adhesion force (Fig. 3 of the main text).

Similar to the adhesion force, the snap-out distance is heavily dependent on the relative humidity. This is expected, because the capillary force dominates the adhesion force on hydrophilic-hydrophilic material combinations and the snap-out distance is also heavily dependent on the same force. As RH is increasing, more water is present on the surface,[13] and the capillary force and bridge formation is enhanced.

Another parameter extracted from the $F(D)$-curves, is the distance to zero force (D_{zf}) and is defined as the retraction distance at which no force is acting on the colloidal probe. The dependence on the RH is shown in Fig. S7. For the hydrophilic-hydrophilic interaction, a small increase in D_{zf} is observed, while for other material combinations D_{zf} remains constant. However, a clear difference in distance is observed between the measurements on CF_x and on the pristine surface. On the latter, the distance to zero force is much smaller, indicating that long range forces are acting on a smaller length scale compared to the CF_x layer. This is in agreement with the higher contact electrification voltage observed on the same layer.

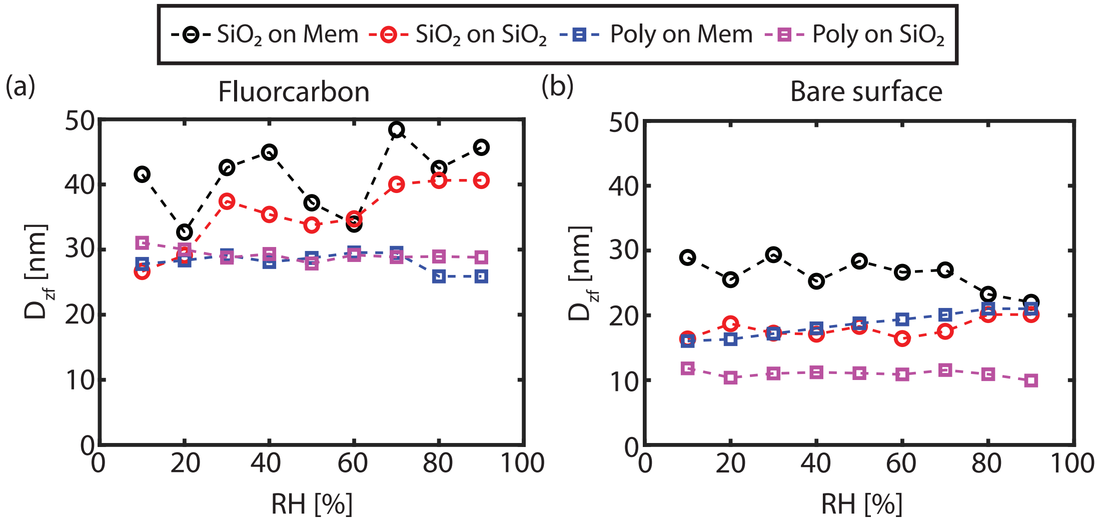

Figure S7: The distance to zero-force (D_{zf}) as a function of the relative humdity for (a) the CF_x coated surface and (b) the pristine surface.

Indentation (δ) has the potential to measure local micromechanical properties of an interface, such as the hardness and elastic modulus.[14] However, the interpretation of the results is complicated by the altering shape of the tip during the experiment and the small

length scales. In Fig. S8 the extracted indentation values are plotted as a function of the RH. No clear trend is observed, but similar to D_{zf} a clear difference is observed between the CF_x coated surfaces and the pristine substrate. A larger indentation is observed on the CF_x layer in agreement with the difference in Young's modulus (see Table S2). In general, also a higher indentation value is found for the measurements performed with a polystyrene colloidal probe (in agreement with the different Young's moduli found in Table S1).

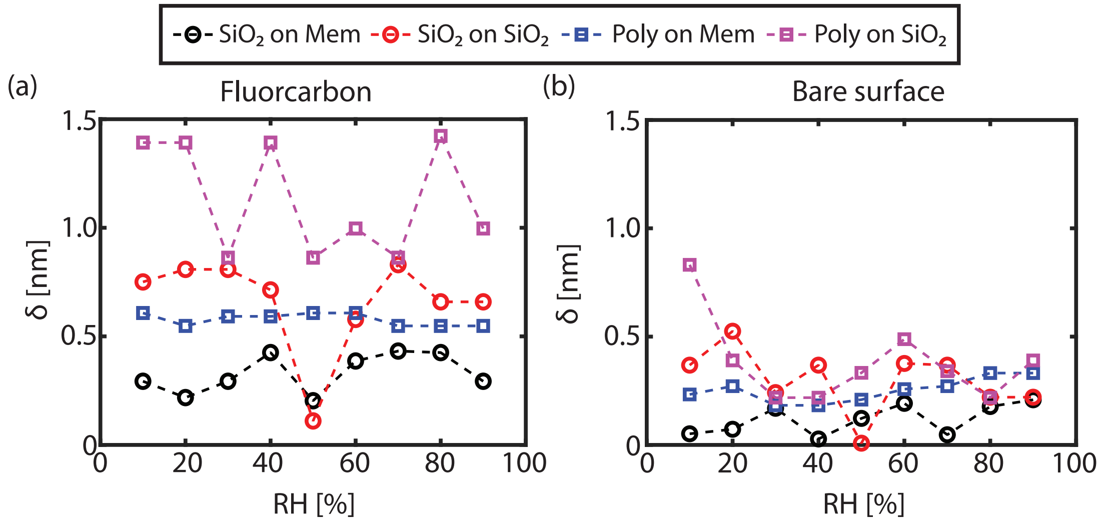

Figure S8: Indentation (δ) as a function of the relative humdity for (a) the CF_x coated surface and (b) the pristine surface.

7 Error Analysis

The uncertainty in the measurements is determined by calculating the standard deviation per measurement point in Figure S9 and Figure S10. Each measurement point in these Figures consists of 30-60 measurements. The adhesion and/or contact electrification voltage is determined from each curve. The standard deviation is visualized as the error bars in Figure S9 and Figure S10. In Figure S9, the error is significantly larger for the hydrophilic-hydrophilic material combination. This is most likely caused by the inhomogeneity in the water layer present on the colloid and the surface. The error contains the standard deviation determined

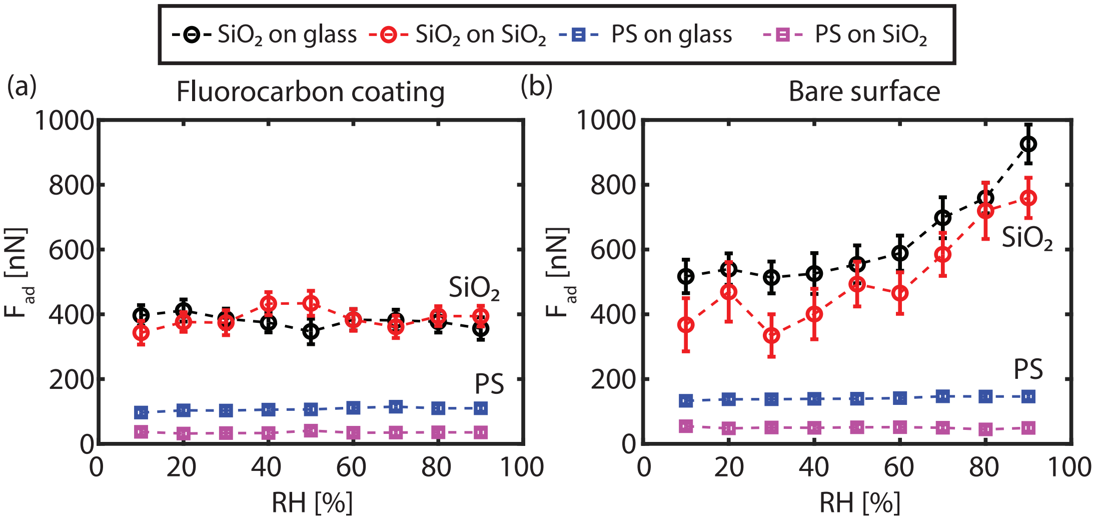

Figure S9: Figure 4 from the main text including error bars.

from the different measurements for the contact electrification voltage. Deviations occurring in the fitting procedure are not incorporated in the error.

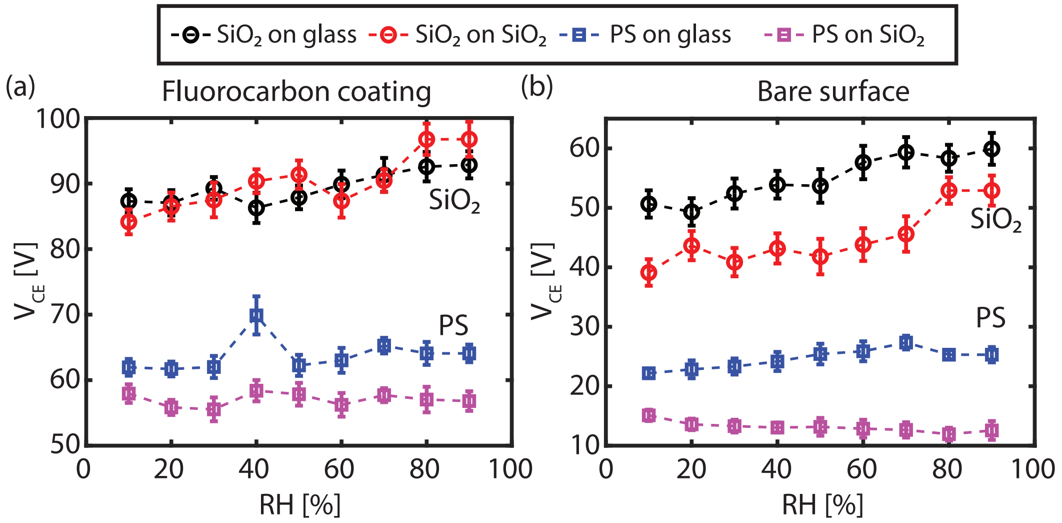

Figure S10: Figure 4 from the main text including error bars.

8 Influence of multiple touches

In several studies, charging of an object was monitored as a function of the number of collisions. In most cases, the object is obtaining charge till the saturation charge level is

reached.[15–17] In Fig. S11 the charge progress is monitored as a function of number of contacts (N_c). For all humidities and approach velocities, no additional charge is accumulated on the colloidal probe and on the surface. This is caused by the speed of the measurement compared to the obtained time constant of charging (Fig. 5b in the main text). During the measurement, the charge is already vanishing into the vapor phase. This process is faster then the measurement method and therefore no additional charging is observed. This agrees with the absence of electrostatic interaction in the approach curves (for instance Fig. 2 in the main text).

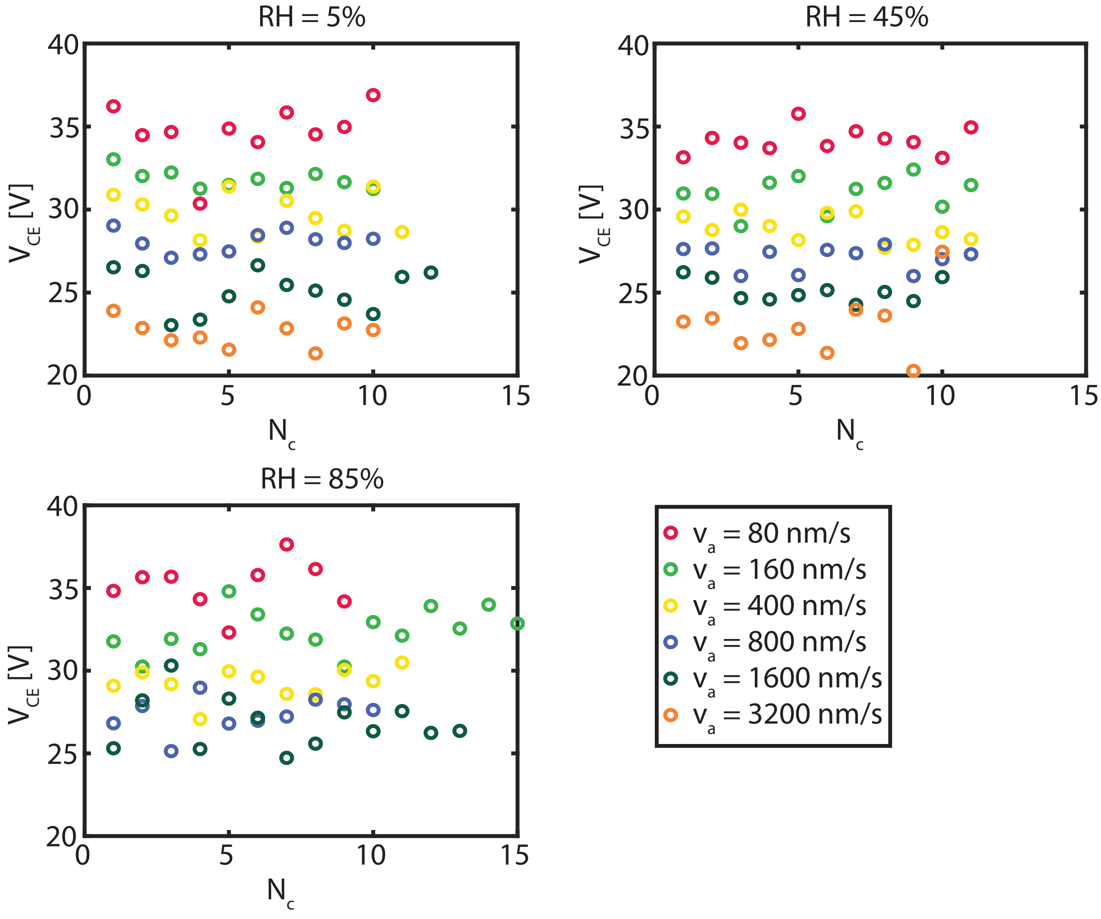

Figure S11: The potential difference (V_{CE}) versus the number of contacts (N_c) for different relative humidities and approach velocities. No charging is observed between the different contact moments.

When the approach velocity is significantly increased, a sign of tribocharging is observed

in the approach curve. An example is shown in Fig. S12. For slow approach velocities (red curve in Fig. S12), the force acting on the cantilever is zero till it snaps into contact. For the $F(D)$-curve measured with a fast approach velocity (blue curve in Fig. S12), an additional force is already interacting with the cantilever. Before the snap-in moment, the measured force is non-zero indicating an interaction between the surface and the cantilever. Because of the fast approach velocity and zero dwell time, the measurement takes approximately 0.125 s, which is faster than the obtained time constant of charging ($\tau_{\mathrm{d}} \approx 0.5$ s). Therefore, a small amount of charge can still be detected. However, the measurement is still too slow to observe the accumulation of charge (see Fig. S11).

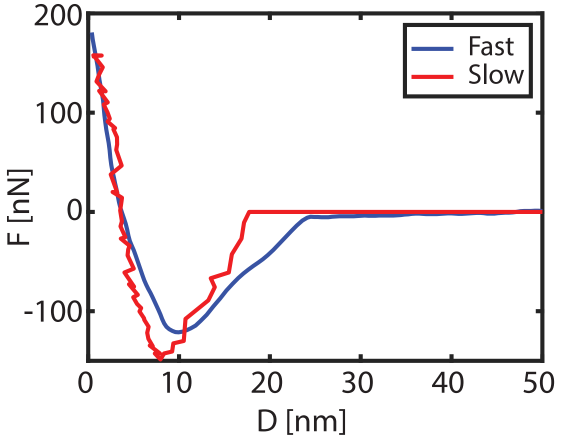

Figure S12: An example of a fast (blue, $v_{\mathrm{a}} = 8000$ nm/s) and a slow (red, $v_{\mathrm{a}} = 160$ nm/s) approach $F(D)$-curve. The fast curve contains a electrostatic component, while in the red curve the electrostatic component is absent.

9 Influence of the approach velocity

The influence of the contact time on the contact electrification voltage is shown in Fig. 5 and discussed in detail in the main text. However, also the approach velocity (v_{a}) is varied. Note that the approach velocity is equal to the retraction velocity discussed in the main text. In Fig. S13, V_{CE} is plotted versus v_{a} for different dwell times. The model described in eq. 6 can be extended further to describe a particle impacting on a plane surface which is a good

approximation for the experiment performed in this study. The contact area A_c is given by

$$A = \pi a^2 \alpha \tag{S3}$$

where

$$\alpha = \left[\frac{5}{4}\pi^2 \rho v^2 (k_p + k_s)\right]^{2/5} \tag{S4}$$

with a the particle radius, v the particle impact velocity, ρ the particle density, $k_{p,s}$ the elasticity parameter of the particle and surface, respectively. The elasticity parameter k is defined as $k = \frac{1-\nu^2}{\pi E}$ where ν is the Poisson ratio and E the Young's modulus. Combining eq. S3 with eq. 6, where $C = \epsilon_0 A Z$ (with Z the effective separation in the order of 1 nm[18]), the contact charge as a function of time and velocity is given by

$$Q = \frac{\pi \epsilon_0 V}{Z}\left[\frac{5}{4}\pi^2 \rho v^2 (k_p + k_s)\right]^{2/5} a^2 (1 - e^{-t_c/\tau_c}) \tag{S5}$$

A weak dependence is expected between the impact velocity and the amount of charge. This is confirmed by the measurements in Fig. S13. For long dwell times ($t_d > 400$ ms), the approach velocity has a small influence on the measured V_{CE} value. For all three cases, a slightly increasing trend is observed, as predicted by eq. S5. Besides the influence of the velocity on the charging process in terms of the contact time, the velocity also affects the speed with which the electrostatic force is measured. When the velocity increases, the retraction curve is measured faster and concomintantly the electrostatic force is measured faster. Consequently, less charge diffused away within the atmosphere in the humidity chamber and a larger V_{CE} is measured.

For small dwell times ($t_d < 400$ ms), a decreasing trend is observed between the contact electrification voltage and the approach velocity. This trend change is caused by the influence of v_a on t_c. As explained in section 4, v_a partly determines the contact time. As the dwell time becomes smaller, the contribution of v_a becomes increasingly more dominant. As v_a is getting larger, the contact time is decreasing and therefore V_{CE} is smaller.

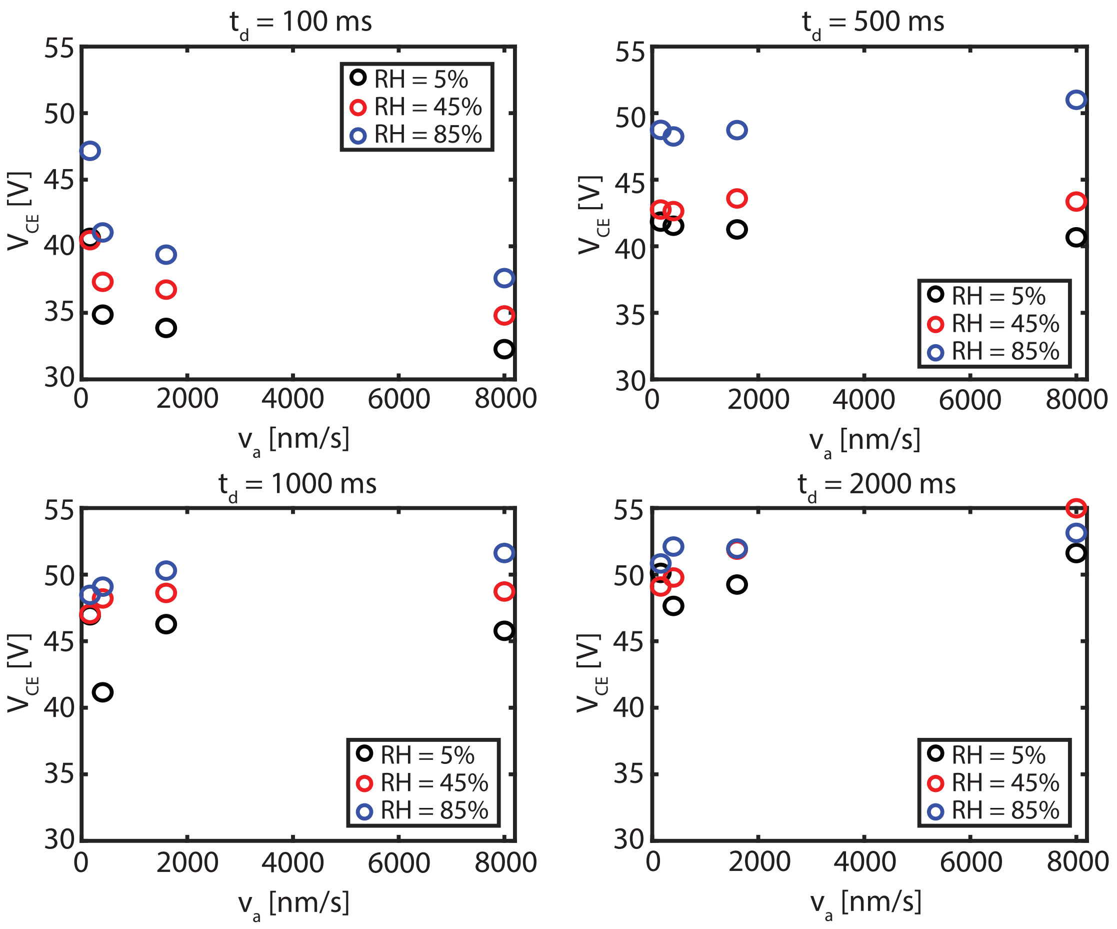

Figure S13: The potential difference (V_{CE}) versus the approach velocity (v_{a}) for different dwell times. When the dwell time is small ($t_{\mathrm{d}} < 400$ ms), the velocity has a clear influence on V_{CE}. When the dwell time is large ($t_{\mathrm{d}} > 400$ ms), the influence of v_{a} is getting smaller.

The influence of v_a is much smaller compared to t_c at high velocities. In Fig. S14 the contact electrification voltage is plotted as a function of the contact time for different approach velocities. Although the dependence on the velocity is changing for different dwell times (Fig. S13), still a clear dependence is observed between V_{CE} and t_c, which is in agreement with the data presented in Fig. 5a.

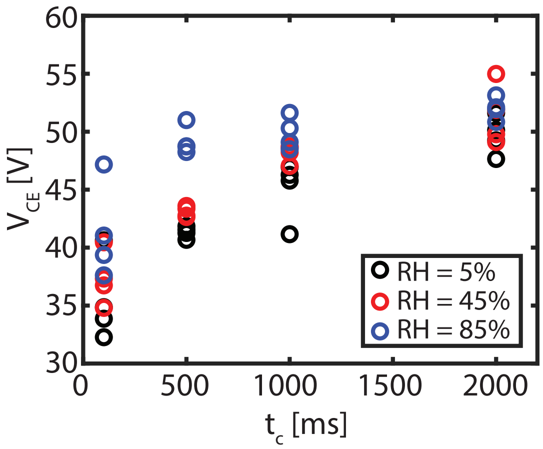

Figure S14: The potential difference (V_{CE}) versus the contact time (t_c) for different relative humidities and approach velocities. A similar trend as in Fig. 5 in the main text is observed for all the different measurements.

References

(1) Hutter, J. L.; Bechhoefer, J. Calibration of atomic-force microscope tips. *Review of Scientific Instruments* **1993**, *64*, 1868–1873.

(2) Wortman, J. J.; Evans, R. A. Young's Modulus, Shear Modulus, and Poisson's Ratio in Silicon and Germanium. *Journal of Applied Physics* **1965**, *36*, 153–156.

(3) Dunlap, W. C.; Watters, R. L. Direct Measurement of the Dielectric Constants of Silicon and Germanium. *Phys. Rev.* **1953**, *92*, 1396–1397.

(4) Jimidar, I. S. M.; Sotthewes, K.; Gardeniers, H.; Desmet, G. Spatial Segregation of Microspheres by Rubbing-Induced Triboelectrification on Patterned Surfaces. *Langmuir* **2020**, *36*, 6793–6800.

(5) Tsaur, S.-L.; Fitch, R. M. Preparation and properties of polystyrene model colloids: II. Effect of surface charge density on coagulation behavior. *Journal of Colloid and Interface Science* **1987**, *115*, 463–471.

(6) Tsai, C.-J.; Pui, D. Y. H.; Liu, B. Y. H. Elastic Flattening and Particle Adhesion. *Aerosol Science and Technology* **1991**, *15*, 239–255.

(7) Israelachvilli, J. *Intermolecular and Surface Forces*; Elsevier Pte Singapore, 2011.

(8) Sotthewes, K.; Bampoulis, P.; Zandvliet, H. J. W.; Lohse, D.; Poelsema, B. Pressure-Induced Melting of Confined Ice. *ACS Nano* **2017**, *11*, 12723–12731, PMID: 29112376.

(9) Birkhölzer, Y. A.; Sotthewes, K.; Gauquelin, N.; Riekehr, L.; Jannis, D.; van der Minne, E.; Bu, Y.; Verbeeck, J.; Zandvliet, H. J. W.; Koster, G.; Rijnders, G. High-Strain-Induced Local Modification of the Electronic Properties of VO2 Thin Films. *ACS Applied Electronic Materials* **2022**, *4*, 6020–6028.

(10) Law, B. M.; Rieutord, F. Electrostatic forces in atomic force microscopy. *Phys. Rev. B* **2002**, *66*, 035402.

(11) Butt, H.-J.; Cappella, B.; Kappl, M. Force measurements with the atomic force microscope: Technique, interpretation and applications. *Surface Science Reports* **2005**, *59*, 1–152.

(12) Jones, R.; Pollock, H. M.; Cleaver, J. A. S.; Hodges, C. S. Adhesion Forces between Glass and Silicon surfaces in Air Studied by AFM: Effects of Relative Humidity, Particle Size, Roughness, and Surface Treatment. *Langmuir* **2002**, *18*, 8045–8055.

(13) Chen, L.; He, X.; Liu, H.; Qian, L.; Kim, S. H. Water Adsorption on Hydrophilic and Hydrophobic Surfaces of Silicon. *J. Phys. Chem. C* **2018**, *122*, 11385–11391.

(14) Monclus, M. A.; Young, T. J.; Di Maio, D. AFM indentation method used for elastic modulus characterization of interfaces and thin layers. *Journal of Materials Science* **2010**, *45*, 3190–3197.

(15) Zhou, Y. S.; Liu, Y.; Zhu, G.; Lin, Z.-H.; Pan, C.; Jing, Q.; Wang, Z. L. In Situ Quantitative Study of Nanoscale Triboelectrification and Patterning. *Nano Letters* **2013**, *13*, 2771–2776, PMID: 23627668.

(16) Lee, V.; James, N. M.; Waitukaitis, S. R.; Jaeger, H. M. Collisional charging of individual submillimeter particles: Using ultrasonic levitation to initiate and track charge transfer. *Phys. Rev. Materials* **2018**, *2*, 035602.

(17) Cruise, R. D.; Hadler, K.; Starr, S. O.; Cilliers, J. J. The effect of particle size and relative humidity on triboelectric charge saturation. *Journal of Physics D: Applied Physics* **2022**, *55*, 185306.

(18) Dahneke, B. The influence of flattening on the adhesion of particles. *Journal of Colloid and Interface Science* **1972**, *40*, 1–13.
